# Communities of gastrointestinal helminths of fish in historically connected habitats: habitat fragmentation effect in a carnivorous catfish *Pelteobagrus fulvidraco *from seven lakes in flood plain of the Yangtze River, China

**DOI:** 10.1186/1756-3305-2-22

**Published:** 2009-04-27

**Authors:** Wen X Li, Pin Nie, Gui T Wang, Wei J Yao

**Affiliations:** 1 State Key Laboratory of Freshwater Ecology and Biotechnology, Institute of Hydrobiology, Chinese Academy of Sciences, Wuhan, Hubei Province 430072, PR China

## Abstract

**Background:**

Habitat fragmentation may result in the reduction of diversity of parasite communities by affecting population size and dispersal pattern of species. In the flood plain of the Yangtze River in China, many lakes, which were once connected with the river, have become isolated since the 1950s from the river by the construction of dams and sluices, with many larger lakes subdivided into smaller ones by road embankments. These artificial barriers have inevitably obstructed the migration of fish between the river and lakes and also among lakes. In this study, the gastrointestinal helminth communities were investigated in a carnivorous fish, the yellowhead catfish *Pelteobagrus fulvidraco*, from two connected and five isolated lakes in the flood plain in order to detect the effect of lake fragmentation on the parasite communities.

**Results:**

A total of 11 species of helminths were recorded in the stomach and intestine of *P. fulvidraco *from seven lakes, including two lakes connected with the Yangtze River, i.e. Poyang and Dongting lakes, and five isolated lakes, i.e. Honghu, Liangzi, Tangxun, Niushan and Baoan lakes. Mean helminth individuals and diversity of helminth communities in Honghu and Dongting lakes was lower than in the other five lakes. The nematode *Procamallanus fulvidraconis *was the dominant species of communities in all the seven lakes. No significant difference in the Shannon-Wiener index was detected between connected lakes (0.48) and isolated lakes (0.50). The similarity of helminth communities between Niushan and Baoan lakes was the highest (0.6708), and the lowest was between Tangxun and Dongting lakes (0.1807). The similarity was low between Dongting and the other lakes, and the similarity decreased with the geographic distance among these lakes. The helminth community in one connected lake, Poyang Lake was clustered with isolated lakes, but the community in Dongting Lake was separated in the tree.

**Conclusion:**

The similarity in the helminth communities of this fish in the flood-plain lakes may be attributed to the historical connection of these habitats and to the completion of the life-cycles of this fish as well as the helminth species within the investigated habitats. The diversity and the digenean majority in the helminth communities can be related to the diet of this fish, and to the lacustrine and macrophytic characters of the habitats. The lake isolation from the river had little detectable effect on the helminth communities of the catfish in flood-plain lakes of the Yangtze River. The low similarities in helminth communities between the Dongting Lake and others may just be a reflection of its unique water environment and anthropogenic alterations or fragmentation in this lake.

## Background

Human activities greatly alter the size, shape, and spatial arrangement of natural habitats, and habitat fragmentation may influence the size of populations and dispersal pattern of individuals among populations [1], thus reducing species richness and abundance [2]. Dam construction can disrupt the connectivity of aquatic ecosystems and impede the abilities of aquatic biota to adapt to changes in environmental conditions [3], and also impact persistence of fish populations [4]. The increased habitat fragmentation and reduced local host population may threaten at least local extinction of parasites [5], especially for autogenic parasites with limited dispersal ability, which have been considered one of the main causes leading to the low number of parasite species [6,7]. However the "rescue effect" from other parasite metapopulations can prevent global extinction [8].

In the flood plain of the Yangtze (Changjiang) River, there were many lakes which were historically connected with the river. Since the 1950s, however, most of them have become isolated from the river due to the construction of dams and sluices, and many larger lakes have been subdivided into smaller ones by hydrological projects, road embankment etc [9]. The construction of these artificial barriers has inevitably obstructed the migration of fish among lakes [10]. The yellowhead catfish *Pelteobagrus fulvidraco *is a common fish species found in the Yangtze River [11], and is residential in being able to sexually reproduce in most of these localities where it occurs, such as in lakes, reservoirs and rivers. In previous research, Li et al. [12] investigated populations of a parasitic nematode in the intestines of *P. fulvidraco *in connected and isolated lakes in the flood plain of the river, but no fragmentation effect was detected at the level of genetic diversity of the nematode populations.

In order to detect if the lake fragmentation has any effect on the helminth communities of fish, the present study was designed to investigate communities of helminths in alimentary tracts of the yellowhead catfish *P. fulvidraco *from two connected lakes and five isolated lakes in the flood plain of the Yangtze River, China.

## Methods

During February 2004, fish samples were collected from 12 sites in seven lakes, with three sites in Poyang lake, two each in Dongting, Honghu, and Liangzi lakes, and one each in Tangxun, Niushan, and Baoan lakes. Poyang and Dongting lakes represent the only two lakes which are still connected with Yangtze River. The distribution of these lakes and sampling sites were given in a previous paper by Li et al. [12]. These lakes are shallow in depth ranging among 1.91 – 6.39 m, and vary in area among 37 – 2933 km^2 ^(Table 1). At least 30 yellowhead catfish were obtained from each sample site. The fork length was measured, and the stomach and intestine of each fish were examined for helminths within 24 h after sampling.

**Table 1:** Features of seven lakes in the flood plain of the Yangtze River, China

Lakes	Longitude	Latitude	Average depth (m)	Area (km^2^)
**Poyang**	E115°49'~116°46'	N28°24'~29°46'	5.10	2933
**Dongting**	E111°53'~113°05'	N28°44'~29°35'	6.39	2432
Niushan	E114°19'~114°29'	N30°23'~30°29'	4.00	40
Honghu	E113°11'~113°28'	N29°38'~29°59'	1.91	344
Baoan	E114°39'~114°46'	N30°12'~30°18'	3.40	48
Tangxun	E114°19'~114°29'	N30°23'~30°29'	1.85	37
Liangzi	E114°21'~114°39'	N30°05'~30°18'	4.16	304

Communities of the gastrointestinal helminths were analysed at the infra- and component levels. Prevalence and abundance, as defined by Bush et al. [13], were calculated for each parasite species. Measures of component community structure are: the total number of helminth species, the Berger-Parker dominance index (*d *= *N*_max_*N*^-1^, in which *N*_max _represents the number of individuals in the most abundant species, and *N *the total number of the species in the community), and the Shannon-Wiener index (*H *= - Σ*P*_i _ln*P*_i_, where *P*_i _is the proportion of the individuals in the *i*th species) which describes the richness and abundance of parasites. Measures of infracommunity structures are: mean number of helminth species per fish, mean number of helminth individuals per fish, and mean Brillouin's index per fish (*B *= (ln*N*! - Σln*n*_i_!) N^-1^, where *n*_i _is the number of individuals in the *i*th species). Indexes are defined and calculated as in Magurran [14]. Similarities between individual fish were compared between lakes using the quantitative percentage similarity index (*P *= Σmin (*P*xi, *P*yi), where *P*xi and *P*yi are the proportions of parasite species *i *in the x and y host population, respectively), which compares similarity of two communities in number of species and parasite individuals as described by Hurlbert [15].

Correlation of lake area and diversity was analysed using correlation matrices. Analysis of variance (ANOVA) was used to examine significant difference in fish fork length among lakes. Significant difference in diversity between connected and isolated lakes was analysed statistically using a *t*-test. Correlation analysis of similarities and mean geographical distance between any two lakes was performed by the Mantel test. Cluster analysis of similarities of helminth communities was conducted by using an unweighted pair-group average method (UPGAM).

## Results

A total of 11 species of helminths including 6 species of digeneans, 3 species of nematodes, 1 species of cestode, and 1 acanthocephalan were found in the stomach and intestine of *P. fulvidraco *from the seven lakes, with their infection levels listed in Table 2. The most prevalent and abundant parasite species was the nematode *Procamallanus fulvidraconis*, which was found in all the lakes and in 74.2% of the fish studied and comprised 69.8% of the total parasite specimens recorded. The digeneans *Genarchopsis goppo *and *Coitocoecum plagiorchis *comprised 13.4%, 6.1% of the total parasite specimens, respectively, and the remaining helminth species less than 5% each.

**Table 2:** Prevalence (%) and mean abundance (± SD) of helminths in *Pelteobagrus fulvidraco *from 7 lakes in the flood plain of the Yangtze River, China

Helminth species	**Poyang**	**Dongting**	Niushan	Honghu	Baoan	Tangxun	Liangzi
*Genarchopsis goppo*	63.3% 2.0 ± 2.4	3.3% 0.1 ± 0.6	63.9% 2.6 ± 4.1	6.4% 0.1 ± 0.8	70% 5.9 ± 7.4	90% 6.0 ± 4.8	28.3% 0.4 ± 0.8
*Orientocreadium siluri*	23.3% 0.5 ± 1.1	21.7% 0.5 ± 1.2	25% 0.8 ± 1.8	15.4% 0.4 ± 2.3	30% 0.43 ± 0.77	6.7% 0.1 ± 0.3	50% 1.7 ± 4.3
*Coitocoecum plagiorchis*	41.1% 2.4 ± 5.0	-	22.2% 0.4 ± 0.8	-	30% 1 ± 1.9	30% 1 ± 2.8	16.7% 0.3 ± 0.7
*Echinoparyphium lingulatum*	12.2% 0.5 ± 1.7	3.3% 0.1 ± 0.2	13.9% 0.3 ± 0.8	3.8% 0.1 ± 0.3	-	13.3% 0.1 ± 0.3	13.3% 0.4 ± 1.5
*Dollfustrema vaneyi*	-	-	8.3% 0.3 ± 1.7	-	-	-	-
*Opisthorchis parasiluri*	-	-	-	-	-	6.7% 0.1 ± 0.5	-
*Procamallanus fulvidraconis*	92.2% 10.2 ± 11.1	40% 3.4 ± 8.2	100% 12.3 ± 9.9	74.4% 6.1 ± 6.2	100% 15.8 ± 9.0	93.3% 7.6 ± 6.7	98.3% 13.0 ± 10.1
*Spinitectus gigi*	2.2% 0.1 ± 0.2	-	5.6% 0.1 ± 0.2	6.4% 0.1 ± 1.0	6.7% 0.1 ± 0.3	-	-
*Camallanus cotti*	1.1% 0.1 ± 0.1	1.7% 0.1 ± 0.1	-	-	3.3% 0.1 ± 0.4	-	-
*Gangesia pseudobagri*	-	3.3% 0.1 ± 0.3	-	11.5% 0.1 ± 0.5	20% 0.3 ± 0.7	40% 1.1 ± 2.2	3.3% 0.1 ± 0.8
*Hebsoma violentum*	-	-	5.6% 0.1 ± 0.2	-	3.3% 0.1 ± 0.2	-	15% 0.4 ± 1.3

There were no significant differences in fish fork length among the lakes (*P *> 0.05). The total number of helminth species ranged among 6 and 8 species in the helminth communities (Table 3). The mean number of helminth species per fish was the highest (2.80) in Tangxun lake, but lower in Dongting and Honghu lakes (0.78 and 1.18). The mean number of helminth individuals per fish was the highest (23.61) in Baoan lake, but lower in Dongting and Honghu lakes (4.20 and 6.90). The Brillouin's index was low, being 0.12 and 0.15 in Dongting and Honghu lakes, the index was greater than 0.4 in the other five lakes. The Shannon-Wiener index was apparently lower in Honghu and Dongting lakes (0.22 and 0.18, respectively) than in the other five lakes (all > 0.5). Therefore, the helminth infra-communities were poorer in the species number and individual number in Dongting and Honghu lakes, and richer in Poyang, Niushan, Baoan, Tangxun and Liangzi lakes. The value of the Berger-Parker dominance index varied from 0.47 to 0.88, with the highest observed in Dongting and Honghu Lakes (0.82 and 0.88), but the dominant species in the seven lakes was the nematode *P. fulvidraconis *(Table 3).

**Table 3:** Characteristics of helminth communities and fish length of *Pelteobagrus fulvidraco *from 7 lakes in the flood plain of the Yangtze River, China

Characteristics	**Poyang**	**Dongting**	Niushan	Honghu	Baoan	Tangxun	Liangzi	**Connected lakes**	Isolated lakes
Sample size	90	60	36	78	30	30	60	150	234
Mean fish length ± SD (cm)	16.12 ± 2.31	17.31 ± 1.72	16.61 ± 1.61	16.53 ± 2.20	17.20 ± 2.11	17.33 ± 2.32	17.53 ± 2.43	16.97 ± 2.37	16.76 ± 2.36
Mean no. of species ± SD	2.37 ± 0.94	0.78 ± 0.88	2.44 ± 1.11	1.18 ± 0.94	2.60 ± 1.1	2.80 ± 1.03	2.60 ± 1.20	1.73 ± 1.20	2.13 ± 1.26
Mean no. of hel ind ± SD ^+^	15.70 ± 11.10	4.20 ± 8.90	16. 80 ± 12.2	6.90 ± 6.72	23.61 ± 12.11	16.00 ± 10.72	17.52 ± 11.74	11.09 ± 11.68	14.47 ± 11.73
Brillouin index ± SD	0.45 ± 0.30	0.12 ± 0.19	0.41 ± 0.31	0.15 ± 0.24	0.51 ± 0.25	0.65 ± 0.20	0.44 ± 0.33	0.36 ± 0.31	0.39 ± 0.32
Total no. of species	8	7	8	6	8	7	8	8	11
Shannon-Wiener index	0.58	0.18	0.53	0.22	0.62	0.83	0.57	0.48 ± 0.40	0.50 ± .041
Berger-Parker index	0.65	0.82	0.74	0.88	0.67	0.47	0.74	0.74	0.70
Dominant species	P.p. *	P.p. *	P.p. *	P.p. *	P.p. *	P.p. *	P.p. *	P.p. *	P.p. *

^+ ^Mean number of helminth individuals

* P.p. represents *Procamallanus fulvidraconis*

There were no significant correlations between lake area and the value of Shannon-Wiener and Brillouin's indexes (*P *> 0.05). Significant difference in the value of Shannon-Wiener index was not found between connected lakes (0.48) and isolated lakes (0.50) (*P *= 0.57); but, a significant difference was detected in the mean number of species and mean number of helminth individuals between connected and isolated lakes. This may, however, reflect the low values observed in one of the two connected lakes and also the only two connected lakes still existing in the flood-plain of the river.

**Figure 1:**
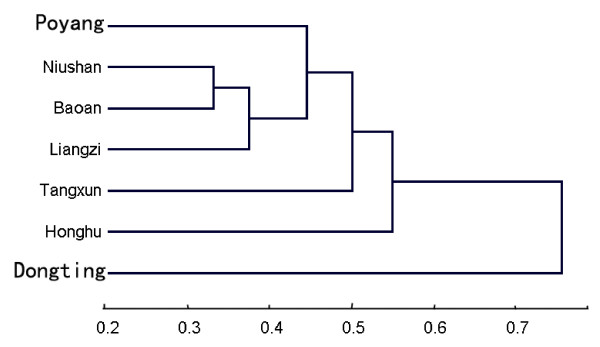
**Clustering tree based on percentage similarity index of between helminth communities in the flood plain of the Yangtze River using unweighted pair-group average method**. Number below the axis indicated the dissimilarity values.

**Figure 2:**
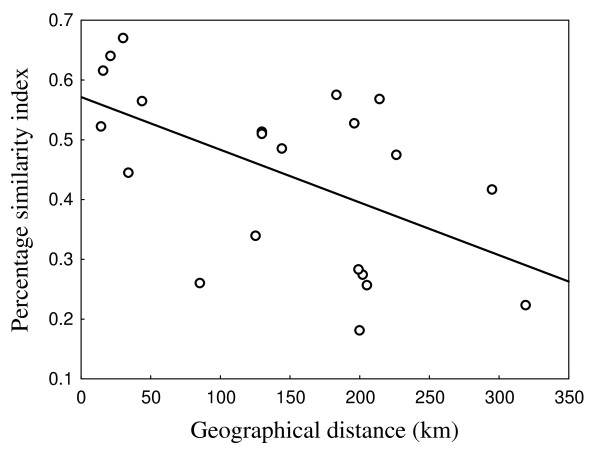
**Relationship between geographical distance and percentage similarity index between each 2 lakes (*R *= - 0.55, *P *< 0.05)**.

The similarity was the highest between Niushan and Baoan lakes (0.67), but lowest between Tangxun and Dongting lakes (0.18). The similarity between Dongting lake and others were very low, all below 0.28. Other comparisons were all above 0.40, except that between Tangxun and Honghu Lakes. From the clustering tree of similarities between helminth communities, Poyang, Niushan, Baoan and Liangzi are clustered together, and then with Tangxun and Honghu lakes; but, Dongting lake was quite separate (Figure 1). The similarity of helminth communities decreased significantly with the geographic distance between lakes (*R *= - 0.55, *P *< 0.05; Figure 2).

## Discussion

It is interesting to note that some characteristics of the gastrointestinal helminth communities, such as the species composition, and the total number of species, were quite similar, or even the same, such as the dominant species, in the investigated connected and isolated flood-plain lakes of the Yangtze River. Furthermore, despite some differences in the mean number of helminth species per fish, the mean number of helminth individuals per fish, and diversity and similarity indexes, the helminth communities of *P. fulvidraco *in isolated lakes were clustered with one connected lake, i.e. Poyang Lake, with the exception of Dongting Lake. This, together with the non-significant difference in the value of Shannon-Wiener index between connected lakes and isolated lakes, may indicate that the isolation from the Yangtze River might have caused little or un-detected effects on major characteristics of the gastrointestinal helminth communities of *P. fulvidraco*.

The low infection level of helminths in Dongting Lake, and its low similarities with other lakes, may just be a reflection of its unique characters. Lakes in the flood-plain of the Yangtze River are in general crescent/oxbow lakes, and were once connected with the river [9]. They are eutrophic, but macrophytic if not influenced recently by urbanization or pollution [9]. Dongting Lake had been vastly reclaimed for agricultural and aquacultural purposes in the last century, with many channels constructed in the lake area in favour of transportation and flood diversion, resulting in a high degree of fragmentation in the lake area although its connection with the Yangtze River is still maintained [16,17]. Most importantly, this lake receives water from four rivers and then flows into the Yangtze River, although Poyang Lake also receives water from a river. The fragmentation, the water composition and probably the overall environment in this lake may account for the observed low infection rate of helminths, and thus the lower values of mean number of species and mean number of helminth individuals, as well as the low similarities observed between Dongting Lake and others, which may then be reflected as a separate tree in the clustering tree. It is nevertheless somewhat disappointing that only two connected lakes were included in the present study, which may also have some effect on the comparison between connected and isolated lakes.

In a study to examine the effect of lake fragmentation on parasite communities of fish, Valtonen et al. [18] also found a high degree of similarities in communities of ecto- and endo- parasites in roach (*Rutilus rutilus*) and perch (*Perca fluviatilis*) from four adjacent lakes, including two isolated lakes in central Finland. They considered that the fish and the parasite faunas may have been originally very similar and minor changes might have occurred over a period of 50 years. This may also be the case for the helminth communities of *P. fulvidraco *in the flood-plain lakes of the Yangtze River, although historical data on the parasites of this fish is not available. The fish can reproduce in lakes and also in rivers, although they may migrate between them, and the helminth species recorded in *P. fulvidraco *can all complete their life cycles in these investigated lakes [19]. The historical connection, the abundance of definitive fish hosts and the completion of life cycles of the helminth species in the flood-plain lakes of the Yangtze River may account for the observed similarity of the gastrointestinal helminth communities in *P. fulvidraco *in the present study. It seems possible that the observed pattern of helminth communities in *P. fulvidraco *in these flood-plain lakes might have been maintained historically, and reflected the historical connection of these localities.

The helminths recorded in the present study are all autogenic. Allogenic parasites have not previously been reported in this catfish [19], although body cavities and other organs of this fish were not examined in the present study. In a previous study, also carried in the flood-plain lakes, the parasites found in the common carp *Cyprinus carpio *were all autogenic [20]. Indeed, allogenic parasites, e.g. allogenic cestodes and/or nematodes are seldom reported in these flood-plain lakes [19], which may be a unique character for the parasite composition of fish in these waters. Autogenic species are considered to have a highly patchy distribution and to be more unpredictable in occurrence than allogenic [7,21], which may lead to the low similarities of parasite communities, as observed in the present study and reported by Esch et al. [7]. Nie et al. [20] also detected a low level of similarity between helminth communities of common carp. However, in other systems, similarities are normally higher; for example the quantitative similarity was high from 0.39 to 0.93 (mean = 0.65 ± 0.03) in whitefish in a group of lakes in Alberta, Canada, and from 0.39 to 0.94 (mean = 0.70 ± 0.04) in lakes in Finland, with the domination of specialists [22,23]. However, in the British Isles, where fish parasite communities have been well-investigated, the level of similarity between component communities is quite variable; but on average is low as determined in natural lakes and reservoirs. These communities were often dominated by generalist parasites, generally acanthocephalans, and are thus unpredictable [7,24,25]. As few data have been gathered in flood-plain lakes, it is far too early to make a predictable suggestion on the pattern of parasite communities in these lakes.

In the flood-plain lakes, several helminth parasites in *P. fulvidraco *may be considered as specialists. Most interestingly, the dominant nematode *Procamallanus fulvidraconis *is a specialist parasite found mostly in this fish, although it has been reported in other species of fish, such as *Silurus asotus *[19]. The higher infection rate of *P. fulvidraconis *observed in these lakes, with the exception of Dongting lake, may be attributed to the lacustrine environment in which zooplanktonic cyclops serving as the intermediate host for the nematode [26] may be abundant, and thus account for, at least to certain extent, the observed similarity between the flood-plain lakes. *Orientocreadium siluri*, *Opisthorchis parasiluri*, *Gangesia pseudobagri*, *Spinitectus gigi *are normally reported from *P. fulvidraco *and other silurid fish, and can then be considered as specialists. The three species, *Genarchopsis goppo*, *Hebsoma violentum *and *Camallanus cotti*, which infect a wide range of fish species, such as cyprinids and silurids may be considered as generalists. *Coitocoecum plagiorchis *and *Dollfustrema vaneyi*, which use carnivorous fish as definitive hosts, infect a wide range of fish species [19,27], may also be recognized as specialists, and *Echinoparyphium lingulatum *was also reported from carnivorous fish [28]. It is thus likely that component communities in *P. fulvidraco *are composed mainly of helminths specific to this fish or silurid fish, and if extended, to carnivorous fish.

The diversity indexes of helminth communities of the catfish were rather high in both isolated and connected lakes. Usually, the richness of helminth communities was related to the diet of host [29]. The yellowhead catfish is carnivorous and feeds mainly on invertebrates, including insect larvae, shrimps and crawfish, and also small fish [30]. Other studies also suggested that helminth communities in carnivorous fish species were generally richer than those in herbivores and omnivores [31]. The mean Brillouin's index of gastrointestinal helminth communities in the carnivorous catfish (0.39) was apparently higher than that (less than 0.16) in another omnivorous European eel *Anguilla anguilla *[32-34], and brown trout *Salmo trutta *[35,36]. Indeed, the Brillouin's index and the species richness of intestinal helminth communities were rather low (from 0 to 0.41 and 1 to 7, respectively) in the common carp *Cyprinus carpio *in flood-plain lakes of the river, which is omnivorous and feeds mainly on invertebrates and plant matters [20]. The majority of digeneans in the component communities can be attributed to the lacustrine and macrophytic environment which may provide habitats for mollusc intermediate hosts and thus transmission of digeneans, although detailed information on molluscan species composition and abundance is not available.

However, the similarity of helminth communities in *P. fulvidraco *reduced with geographical distances among lakes. The similarity of parasite communities decreasing with geographical distance is a pattern of many reported fish parasite communities [37-39]. Geographical distance may influence the probability of parasite exchanges [22,38], and there may be several explanations for such reduced similarity. The historical connection may be attributed to the observed similarity of helminth communities, but the degrees of stocking fishery in these lakes, and possible factors such flooding which may occur frequently in this flood-plain [9] may cause the variation of the helminth community similarities, or increase the similarity between adjacent lakes. Overall, the island biogeography theory may provide some explanation for the similarities of helminth communities between lakes. In particular, the lower similarity between Dongting Lake and the others might have influenced substantially the reduced similarity, and it may not be the only matter of distance, but the uniqueness of Dongting Lake as described above.

In addition, populations of these helminth species may be large enough to maintain population structure, and thus reduce the difference at both the population and community levels. Li et al. [12] reported that the nematode *P. fulvidraconis*, which dominated the helminth communities of *P. fulvidraco *in flood-plain lakes, showed no genetic difference at the level of populations also in these lakes. It may also be possible that helminths can reinvade from other local populations of fish, with the movement of intermediate and definitive hosts. Therefore, lake isolation from the Yangtze River has hardly any effect on helminth communities of *P. fulvidraco *in such a short period of about half a century.

Other factors, such as trophic status have been suggested to have some effect on helminth communities of fish. Historical and recent studies have all shown that helminth component community in fish was associated with physicochemical characteristics and thus productivity of lakes [18,23,40]. Eutrophication has always been a problem in flood-plain lakes of the Yangtze River, but the increase in trophic level and artificial stocking in some lakes in the flood-plain have inevitably destroyed the submerged plantation, leading to the conversion of original, so-called macrophytic lakes to algal lakes in which the carnivorous catfish *Pelteobagrus fulvidraco *has almost disappeared [41]. So, the effect of eutrophication on helminth communities of fish in these lakes may be interpreted cautiously or differently as generally recognized.

## Competing interests

The authors declare that they have no competing interests.

## Authors' contributions

WXL conducted the field work and data analysis and drafted the manuscript. PN generated the research idea and finalized the manuscript. GTW contributed to the research plan. WJY participated in the field work. All authors read and approved the final manuscript.

## References

[B1] Fahrig L, Merriam HG (1994). Conservation of fragmented population. Conservation Biology.

[B2] Gibb H, Hochuli DF (2002). Habitat fragmentation in an urban environment: large and small fragments support different arthropod assemblages. Biological Conservation.

[B3] Pringle CM, Freeman MC, Freeman BJ (2000). Regional effects of hydrologic alterations on riverine macrobiota in the New World: tropical-temperate comparisons. BioSience.

[B4] Morita K, Yamamoto S (2002). Effects of habitat fragmentation by damming on the persistence of stream-dwelling charr populations. Conservation Biology.

[B5] Dobson AP, Pacala SW (1992). The parasites of *Anolis *lizards of the northern Lesser Antilles. II. The structure of the parasite community. Oecologia.

[B6] Kennedy CR, Bush AO, Aho JM (1986). Patterns in helminth communities: why are birds and fish different. Parasitology.

[B7] Esch GW, Kennedy CR, Bush AO, Aho JM (1988). Patterns in helminth communities in freshwater fish in Great Britain: alternative strategies for colonization. Parasitology.

[B8] Bush AO, Kennedy CR (1994). Host fragmentation and helminth parasites: hedging your bets against extinction. International Journal for Parasitology.

[B9] Wang S, Dou H (1998). Chinese lakes.

[B10] Chang JB, Cao WX (1999). Fishery significance of the river-communicating lakes and strategies for the management of fish resources. Resources and Environment in the Yangtze River.

[B11] Cheng Q, Zheng B (1987). Systematic synopsis of Chinese fishes.

[B12] Li WX, Wang GT, Nie P (2008). Genetic variation of fish parasite populations in historically connected habitats: undetected habitat fragmentation effect on populations of the nematode *Procamallanus fulvidraconis *in the catfish *Pelteobagrus fulvidraco*. J Parasitol.

[B13] Bush AO, Lafferty KD, Lotz JM, Shostak AW (1997). Parasitology meets ecology on its own terms: Margolis et al. revisited. Journal of Parasitology.

[B14] Magurran AE (1988). Ecological Diversity and its Measurement.

[B15] Hurlbert SH (1978). The measurement of niche overlap and some relatives. Ecology.

[B16] Xiong JX (2008). Integrity of spatial structure of wetland landscape in west Dongting Lake and its optimization. Wetland Science & Management.

[B17] Xiong JX, Wu NF (2008). Analysis of spatial structural integrity of wetland landscape in the east Dongting Lake. Environmental Science & Management.

[B18] Valtonen ET, Holmes JC, Koskivaara M (1997). Eutrophication, pollution, and fragmentation: effects on parasite communities in roach (*Rutilus rutilus*) and perch (*Perca fluviatilis*) in four lakes in central Finland. Canadian Journal of Fisheries Aquatic Sciences.

[B19] Chen CI, Eds (1973). An illustrated guide to the fish disease and causative pathogenic fauna and flora in the Hupei Province.

[B20] Nie P, Yao WJ, Gao Q, Wang GT, Zhang YA (1999). Diversity of intestinal helminth communities of carp from six lakes in the flood plain of the Yangtze River, China. Journal of Fish Biology.

[B21] Esch G, Fernández J (1993). A functional biology of parasitism: Ecological and evolutionary implications.

[B22] Karvonen A, Valtonen ET (2004). Helminth assemblages of whitefish (*Coregonus lavaretus*) in interconnected lakes: similarity as a function of species specific parasites and geographical separation. Journal of Parasitology.

[B23] Goater CP, Baldwin RE, Scrimgeour GJ (2005). Physico-chemical determinants of helminth component community structure in whitefish (*Coregonus clupeaformes*) from adjacent lakes in Northern Alberta, Canada. Parasitology.

[B24] Kennedy CR (1990). Helminth communities in freshwater fish: structural communities or stochastic assemblages?. Parasite Communities: Patterns and Processes.

[B25] Hartvigsen R, Kennedy CR (1993). Patterns in the composition and richness of helminth communities in brown trout, *Salmo trutta*, in a group of reservoirs. Journal of Fish Biology.

[B26] Li HC (1935). The taxonomy and early development of *Procamallanus fulvidraconis *n. sp. Journal of Parasitology.

[B27] Wang GT (2003). A note on the monthly changes of *Dollfustrema Vaneyi *in the digestive tract of the mandarin fish, *Siniperca chuatsi*. Acta Hydrobiological Sinica.

[B28] Wang PQ (1984). Some digenetic treamatodes from fishes in Fujian province, China. Acta Zootaxonomica Sinica.

[B29] Dogiel VA (1964). General Parasitology (English translation).

[B30] Du JR (1963). A study on the ingredient and emergence rate of the food and the reproduction of *Pseudobagrus fulvidraco *in Liangzi Lake. Chinese Journal of Zoology.

[B31] Pérez-Ponce de León G, García-Prieto L, León-Règagnon V, Choudhury A (2000). Helminth communities of native and introduced fishes in Lake Pátzcuaro, Michoacán, México. Journal of Fish Biology.

[B32] Kennedy CR (1993). The dynamics of intestinal helminth communities in eels *Anguilla anguilla *in a small stream: long-term changes in richness and structure. Parasitology.

[B33] Sures B, Knopf K, Wurtz J, Hirt J (1999). Richness and diversity of parasite communities in European eels *Anguilla anguilla *of the River Rhine, Germany, with special reference to helminth parasites. Parasitology.

[B34] Kristmundsson A, Helgason S (2007). Parasite communities of eels *Anguilla anguilla *in freshwater and marine habitats in Iceland in comparison with other parasite communities of eels in Europe. Folia Parasitologica.

[B35] Molloy S, Holland C, Poole R (1995). Metazoan parasite community structure in brown trout from two lakes in western Ireland. Journal of Helminthology.

[B36] Kennedy CR, Hartvigsen RA (2000). Richness and diversity of intestinal metazoan communities in brown trout *Salmo trutta *compared to those of eels *Anguilla anguilla *in their European heartlands. Parasitology.

[B37] Poulin R, Morand S (1999). Geographical distances and the similarity among parasite communities of conspecific host populations. Parasitology.

[B38] Poulin R (2003). The decay of similarity with geographical distance in parasite communities of vertebrate hosts. Journal of Biogeography.

[B39] Oliva1 ME, González MT (2005). The decay of similarity over geographical distance in parasite communities of marine fishes. Journal of Biogeography.

[B40] Wisniewski WL (1958). Characterization of the parasite fauna of an eutrophic lake. Acta Parasitologica Polonica.

[B41] Yan G, Ma J, Qiu D, Wu Z (1997). Succession and species replacement of aquatic plant community in East lake. Acta Phytoecologica Sinica.

